# Concurrent chronic kidney disease in patients with inflammatory bowel disease, a systematic review and meta-analysis

**DOI:** 10.3389/fmed.2024.1485087

**Published:** 2024-10-03

**Authors:** Xiaoping Han, Zifeng Xu, Yu Chang, Hongyan Li, Sileng Hu, Shiyu Chang, Yue Liu, Chanjiao Yu, Tongyu Tang, Yuqin Li

**Affiliations:** ^1^Department of Gastroenterology, The First Hospital of Jilin University, Changchun, China; ^2^Norman Bethune Health Science Center, Jilin University, Changchun, China

**Keywords:** chronic kidney disease, inflammatory bowel disease, meta-analysis, Crohn disease, ulcerative colitis

## Abstract

**Introduction:**

Inflammatory bowel disease (IBD) is a multi-organ autoimmune disease that commonly affects the gastrointestinal tract, but can also affect other organs throughout the body. Less is known, however, about kidney involvement in IBD. Although IBD has been associated with chronic kidney disease (CKD) and end-stage renal disease (ESRD), these results have been inconsistent. The present study analyzed the prevalence of concurrent CKD and ESRD in patients with IBD.

**Methods:**

PubMed, Cochrane, Web of Science, and Embase were searched for studies published through October 2023 on IBD patients with concurrent CKD or ESRD. Outcomes included the incidence rates and odds ratios (OR) of concurrent CKD and ESRD in IBD patients. The quality of included studies was assessed using the Newcastle-Ottawa Scale, and sequential sensitivity was analyzed. Publication bias was evaluated using Egger’s test.

**Results:**

Nine studies were included in this meta-analysis. The combined results of eight studies, which included 239,042 IBD patients, showed that the prevalence of CKD in IBD patients was 5% (95% confidence interval [CI]: 1–9%). The combined results of two studies, which included 40,341 IBD patients, showed that the prevalence of ESRD in IBD patients was 0.2% (95% CI: −0.08–0.12%). The combined results of six case–control studies reported that the risk of CKD was significantly higher in patients with than without IBD (OR 1.36, 95% CI: 1.08–1.70, *p* = 0.008).

**Conclusion:**

Although studies have shown an increased risk of CKD in IBD, due to the small number of included studies and high heterogeneity across studies, it is not enough to definitively conclude that CKD is more common in patients with IBD. But patients with IBD should be regularly monitored for CKD.

**Systematic review registration:**

https://www.crd.york.ac.uk/PROSPERO/.

## 1 Introduction

Inflammatory bowel disease (IBD), consisting mainly of Crohn’s disease (CD) and ulcerative colitis (UC), is characterized by chronic recurrent intestinal inflammation. The pathogenesis of IBD is thought to involve complex interactions among genetic, environmental, and microbial factors, as well as immune responses (1). Traditionally, the incidence rate of IBD is highest in North America and Western Europe. Many cohort studies have shown that the incidence of IBD in these geographic areas increased significantly during the latter half of the 20th century, as well as increasing in populations of emerging nations, including in Asian countries (2).

IBD is a systemic disease that not only affects the gastrointestinal tract but also involves many extraintestinal organs. The quality of life of IBD patients may be significantly influenced by these extraintestinal manifestations (EIMs). Up to 50% of IBD patients have been reported to develop at least one EIM affecting various systems throughout the body (3). EIMs in patients with CD and UC most commonly involve the musculoskeletal system (e.g., peripheral and axial arthritis, enthesitis), the skin (e.g., pyoderma gangrenosum, erythema nodosum, Sweet’s syndrome, aphthous stomatitis), the hepatobiliary system (e.g., primary sclerosing cholangitis), and the eyes (e.g., episcleritis, anterior uveitis, iritis) (4).

Although there are limited data on renal manifestations in IBD patients, up to 5–15% of adult IBD patients may develop chronic kidney disease (CKD) over time (5). CKD has been defined by the Kidney Disease: Improving Global Outcomes (KDIGO) and the National Kidney Foundation (NKF) Kidney Disease Outcomes Quality Initiative (K/DOQI) guidelines as kidney damage or a glomerular filtration rate (GFR) < 60 mL/min/1.73 m^2^ for more than 3 months. These guidelines have defined renal impairment as functional or structural abnormalities of the kidney (6, 7).

The pathophysiology of renal involvement in patients with IBD is complex and not yet fully understood, possibly leading to various renal disorders affecting the glomeruli and/or tubular structure. Furthermore, medications used to treat IBD may be potentially nephrotoxic, and metabolic complications caused by the disease itself may further lead to renal damage (5).

Important predictors of renal failure include age at diagnosis of kidney disease and the chronicity of the disease in kidney biopsies. Overall survival was found to be significantly better in patients who did than did not experience partial or complete resolution of kidney disease, and those who did than did not show stabilization or improvement of renal function (8).

The aim of this study was to quantify the prevalence of CKD and end-stage renal disease (ESRD) in patients with IBD and to assess their associations. Early identification of these renal diseases may improve long-term outcomes in these patients.

## 2 Methods

### 2.1 Literature search and selection of studies

This study follows the Preferred Reporting Items for Systematic Reviews and Meta-Analysis (PRISMA) statement (9). The PubMed, Cochrane, Web of Science, and Embase databases were searched using relevant keywords for studies published through October 2023 on IBD patients with concurrent CKD or ESRD (Supplementary Table S1). Studies were included if they (a) reported the occurrence of CKD/ESRD in IBD patients and (b) were original studies written in English and available in full text. Studies were excluded if (a) study data were missing; (b) they duplicated data or papers; (c) were case reports, letters, editorials, or meta-analyses that did not include original data, or (d) were conference abstracts or review articles. The references cited by included articles were also reviewed to ensure study comprehensiveness and accuracy. If different publications provided data from the same population, only the latest or most comprehensive study was included.

### 2.2 Data extraction

The literature screening process was divided into three steps. Following the initial literature search by one researcher, duplicate papers were removed using EndNote X9 software, followed by manual elimination of any remaining duplicates. A second researcher subsequently read the titles and abstracts of the selected studies, identifying studies that potentially met the inclusion criteria. A third researcher subsequently read the full text of the selected studies, identifying those that met the original criteria. Data were extracted from these studies using a pre-designed standard set of parameters, which included author names, year of publication, country, study period, study design, number of IBD patients (including CD and UC), number of CKD/ESRD patients, and number of control group participants. Any disagreements were resolved through discussion with a fourth researcher.

### 2.3 Quality assessment

The quality of included studies was assessed using the Newcastle-Ottawa Scale (NOS), and scores of 7–9 indicating high-quality studies (10). Two researchers independently assessed the quality and level of evidence of eligible studies, with any differences resolved through discussion with a third researcher.

### 2.4 Statistical analysis

All data were analyzed using Stata software version 18.0. A random-effects model was used to calculate the prevalence of CKD/ESRD and its 95% confidence interval [CI]. The random (M-H heterogeneity) model was applied to estimate the odds ratio (OR) and 95% CI for the occurrence of CKD/ESRD in IBD patients. Study heterogeneity was evaluated using the *I*^2^ test. A random effects model was used when there was high heterogeneity among studies (*I*^2^ > 50%). whereas a fixed effects model was used when there was low heterogeneity among studies (*I*^2^ ≤ 50%). Heterogeneity was assessed by sequential sensitivity analysis, in which one study was sequentially removed in each scenario. Publication bias was analyzed using Egger’s test (11).

## 3 Results

### 3.1 Study selection and quality evaluation

**Figure 1:**
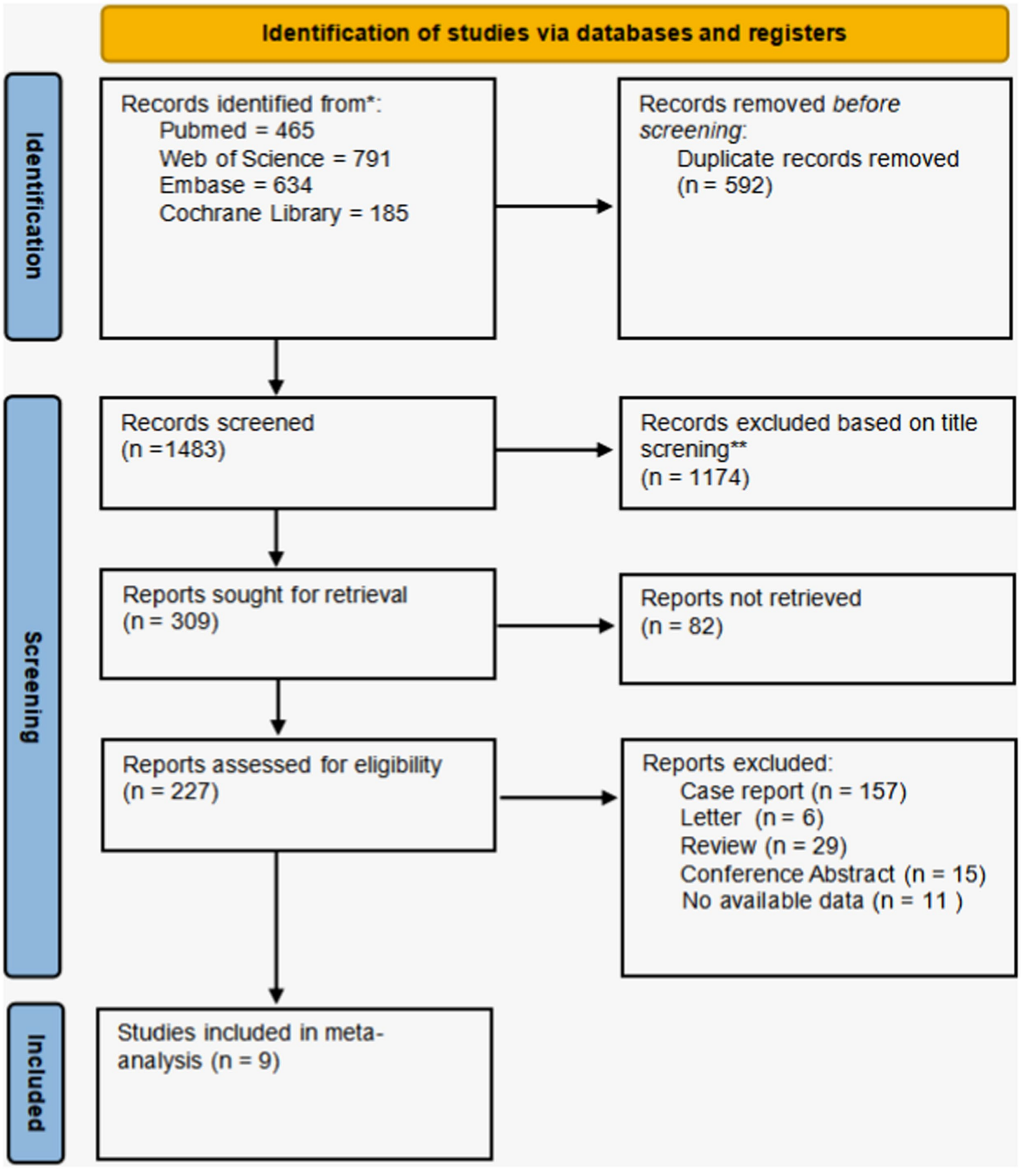
Flow diagram of systematic search and selection.

The original literature search identified a total of 2075 studies, 465 from PubMed, 634 from Embase, 791 from the Web of Science, and 185 from the Cochrane database. Of these, 592 duplicate studies were removed using EndNote X9 and manual screening, and 1,174 irrelevant studies were excluded by reviewing titles and abstracts. Of the 309 remaining studies, 82 could not be retrieved and were therefore excluded. Among the remaining 227 studies, 157 case reports, six letters, 29 reviews, 15 conference abstracts, and 11 studies with unavailable data were excluded, resulting in nine studies included in the meta-analysis. The flowchart of the search results and study selection process is shown in Figure 1. The main characteristics and quality assessment of the included studies are presented in Table 1. All nine included studies were of high quality.

**Table 1:** Characteristics and quality evaluation of included studies.

Study	Study design	Region	Period	Female (%)	IBD				Control			Score
					Total		Events		Total		Events	
Vajravelu 2020	Case–control study	The United Kingdom	NA	8,856 (49.7)	IBD	17,807	CKD	910	Non-IBD	63,466	2,224	9
Bernstein 2005	Case–control study	Manitobans	1984–2003	NA	IBD	8,072	CKD	135	Non-IBD	80,489	659	8
					CD	4,193		76		41,815	326	
					UC	3,879		59		38,674	333	
Yang 2023	Case–control study	Stockholm, Sweden	2006–2018	NA	IBD	9,876	CKD	260	Non-IBD	1,668,651	56,867	8
Zheng 2023	Case–control study	US	2016–2018	106,137 (56.4)	IBD	188,059	CKD	24,274	Non-IBD	188,059	22,802	8
				68,106 (57.9)	CD	117,631		15,177		117,631	13,163	
				38,031 (54.0)	UC	70,428		9,097		70,428	9,639	
Bernstein 2021	Case–control study	Manitobans	1984–2018	53	IBD	9,247	CKD	139	Non-IBD	85,691	817	7
				57	CD	4,253		NA		NA	NA	
				50	UC	4,994		NA		NA	NA	
Park 2018	Case–control study	South Korea	2010–2013	15,132 (40.0)	IBD	38,812	ESRD	79	Non-IBD	116,436	166	9
				3,768 (29.9)	CD	12,585		31		37,755	24	
				11,364 (43.3)	UC	26,227		48		78,681	142	
Liu 2023	Case–control study	England, Wales, and Scotland	2010–2022	54	IBD	4,201	CKD	217	Non-IBD	413,101	13,347	8
				56.7	CD	1,261		70		NA	NA	
				50.9	UC	2,940		147		NA	NA	
Elseviers 2004	Cohort study	Europe	NA	55	IBD	1,529	CKD	13	/			8
							ESRD	2				
Lewis 2013	Cohort study	USA	2009–2010	116 (46.2)	IBD	251	CKD	26	/			7
					CD	166		NA				
					UC	85		NA				

### 3.2 Prevalence of CKD and ESRD in IBD patients

**Figure 2:**
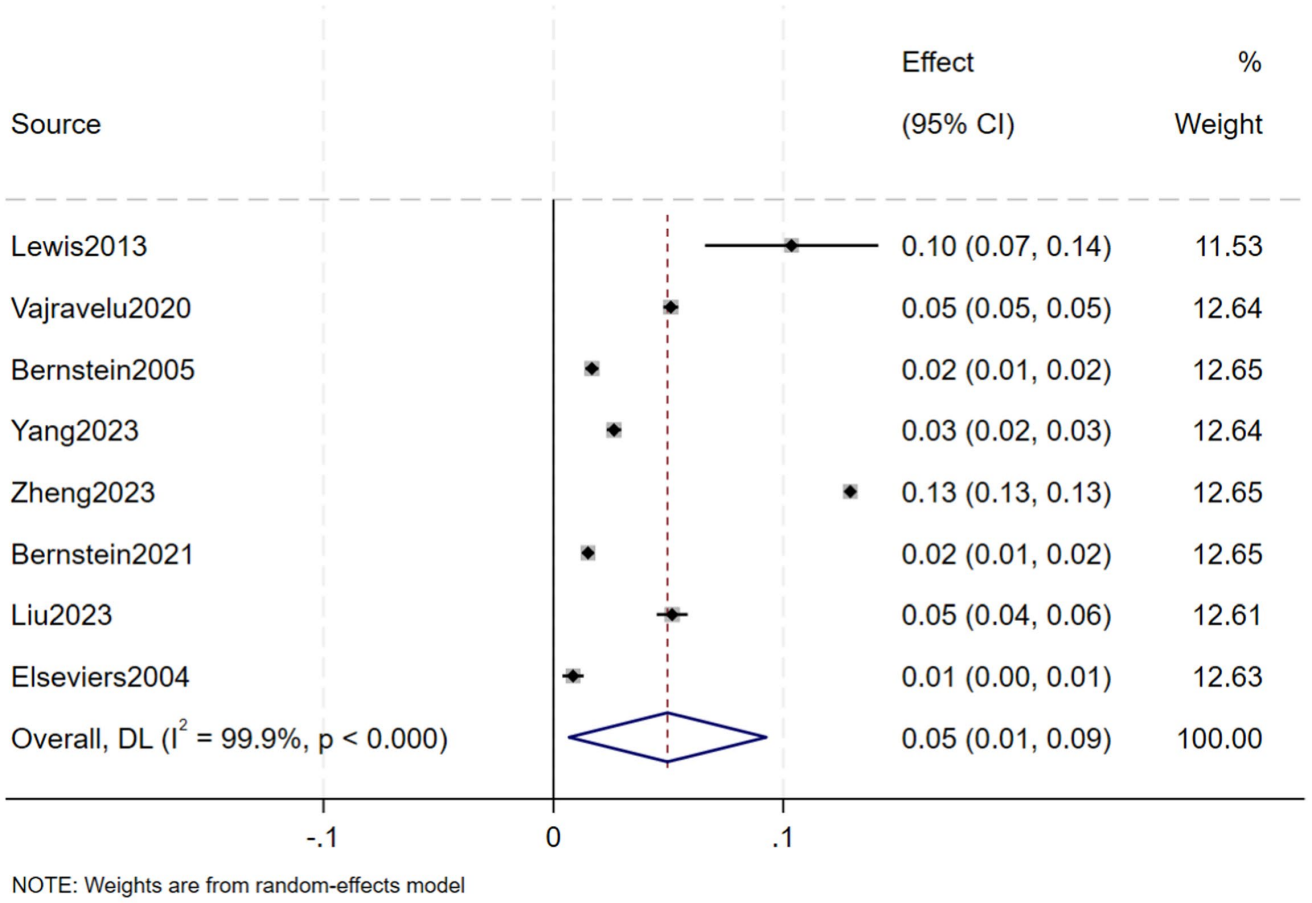
Prevalence of chronic kidney disease in patients with inflammatory bowel disease.

**Figure 3:**
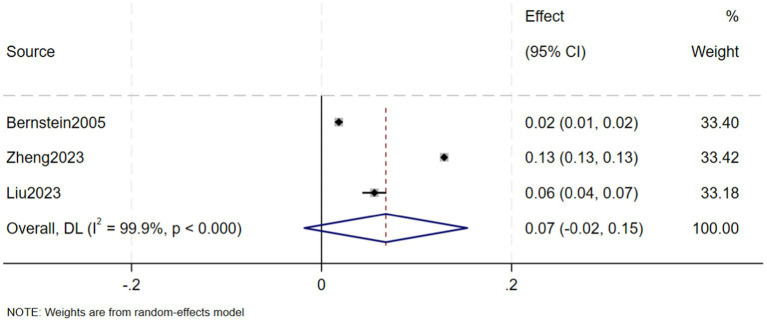
Prevalence of chronic kidney disease in patients with Crohn disease.

**Figure 4:**
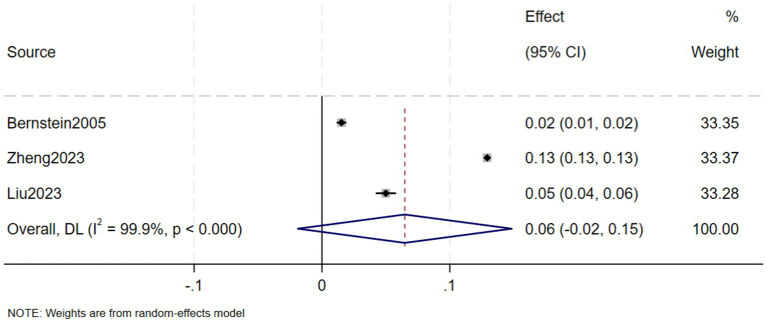
Prevalence of chronic kidney disease in patients with ulcerative colitis.

**Figure 5:**
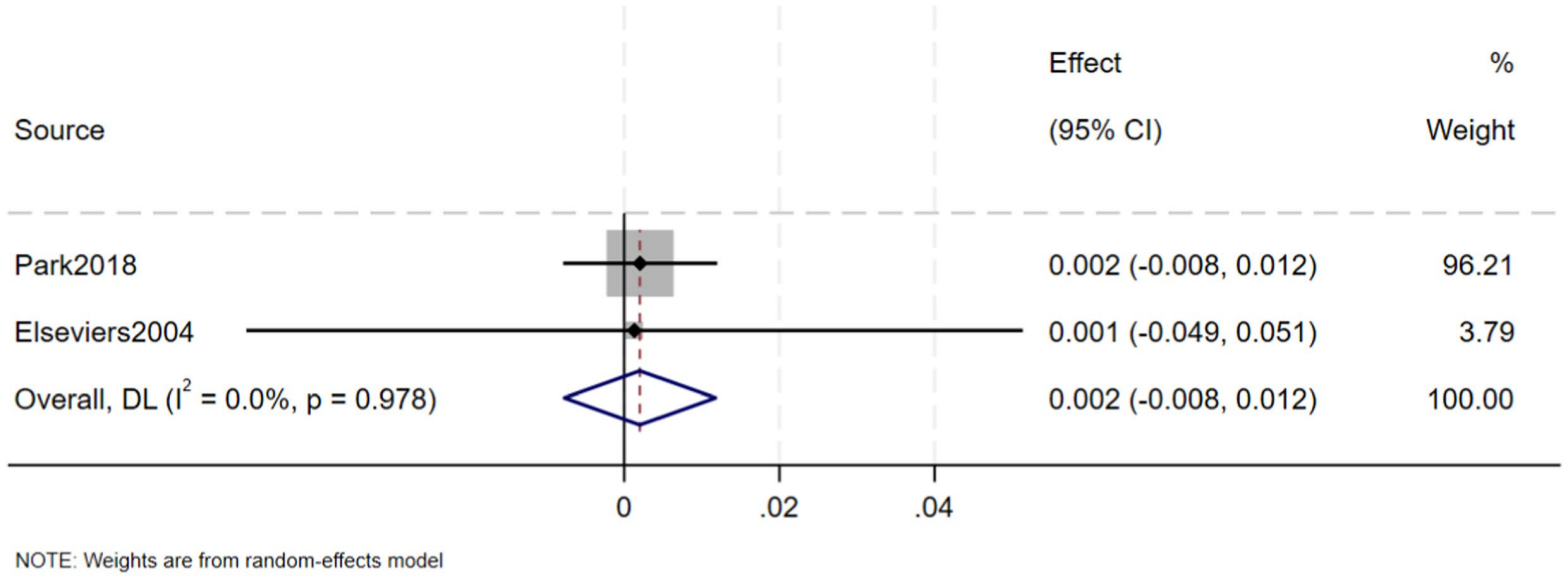
Prevalence of end-stage renal disease in patients with inflammatory bowel disease.

A total of eight studies, involving 239,042 patients with IBD, reported that the prevalence of CKD in in IBD patients was 5% (95% CI: 1–9%, *I*^2^ = 99.9%, heterogeneity *p* < 0.000) (Figure 2). Three of these studies reported the prevalence of CKD separately in CD and UC patients, finding that the prevalence of CKD in 123,085 CD patients was 7% (95% CI: −2–15%, *I*^2^ = 99.9%, heterogeneity *p* < 0.000) (Figure 3) and the prevalence of CKD in 77,247 UC patients was 6% (95% CI: −2–15%, *I*^2^ = 99.9%, heterogeneity *p* < 0.000) (Figure 4). Two studies, involving 40,341 patients with IBD, reported that the prevalence of ESRD was 0.2% (95% CI: −0.08–0.12%, *I*^2^ = 0.0%, heterogeneity *p* = 0.987) (Figure 5). Considering the single-group rate as a descriptive result, not a comparison of differences, sensitivity and publication bias analyses were not performed.

### 3.3 Association between IBD and CKD

**Figure 6:**
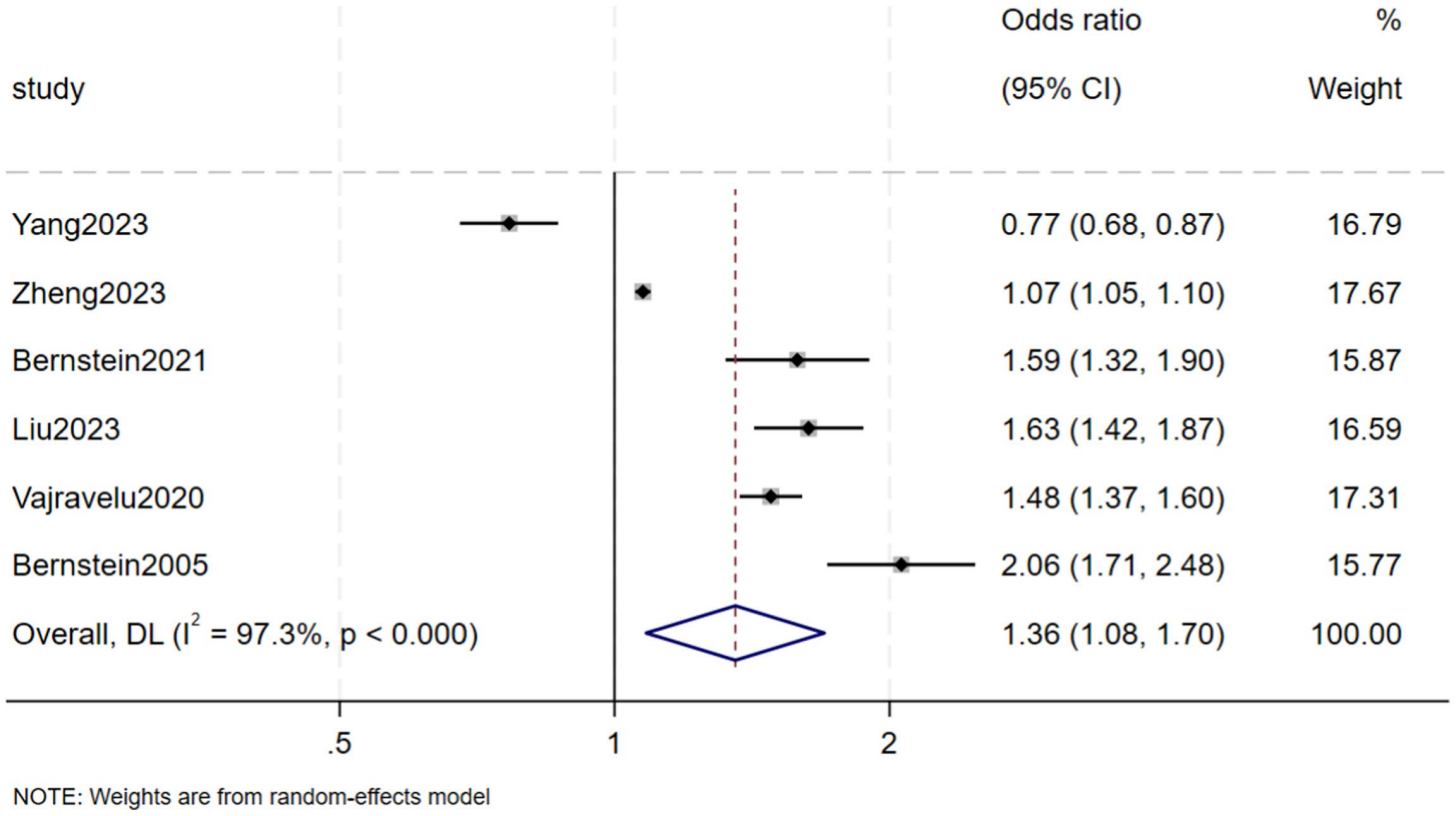
Risk of chronic kidney disease among the inflammatory bowel disease patients.

**Figure 7:**
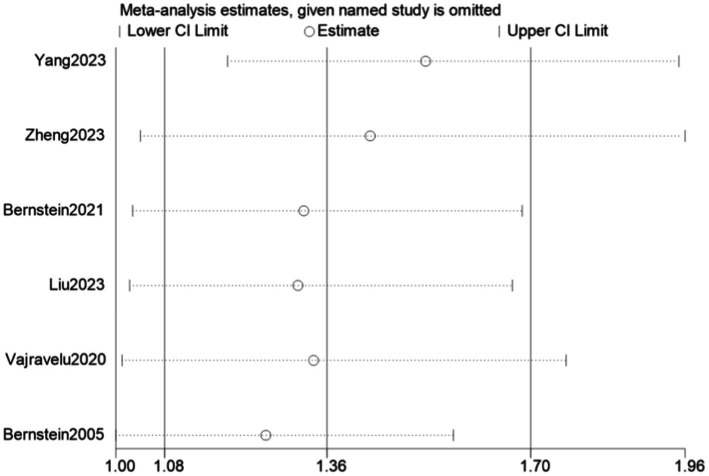
Sensitivity analysis.

**Figure 8:**
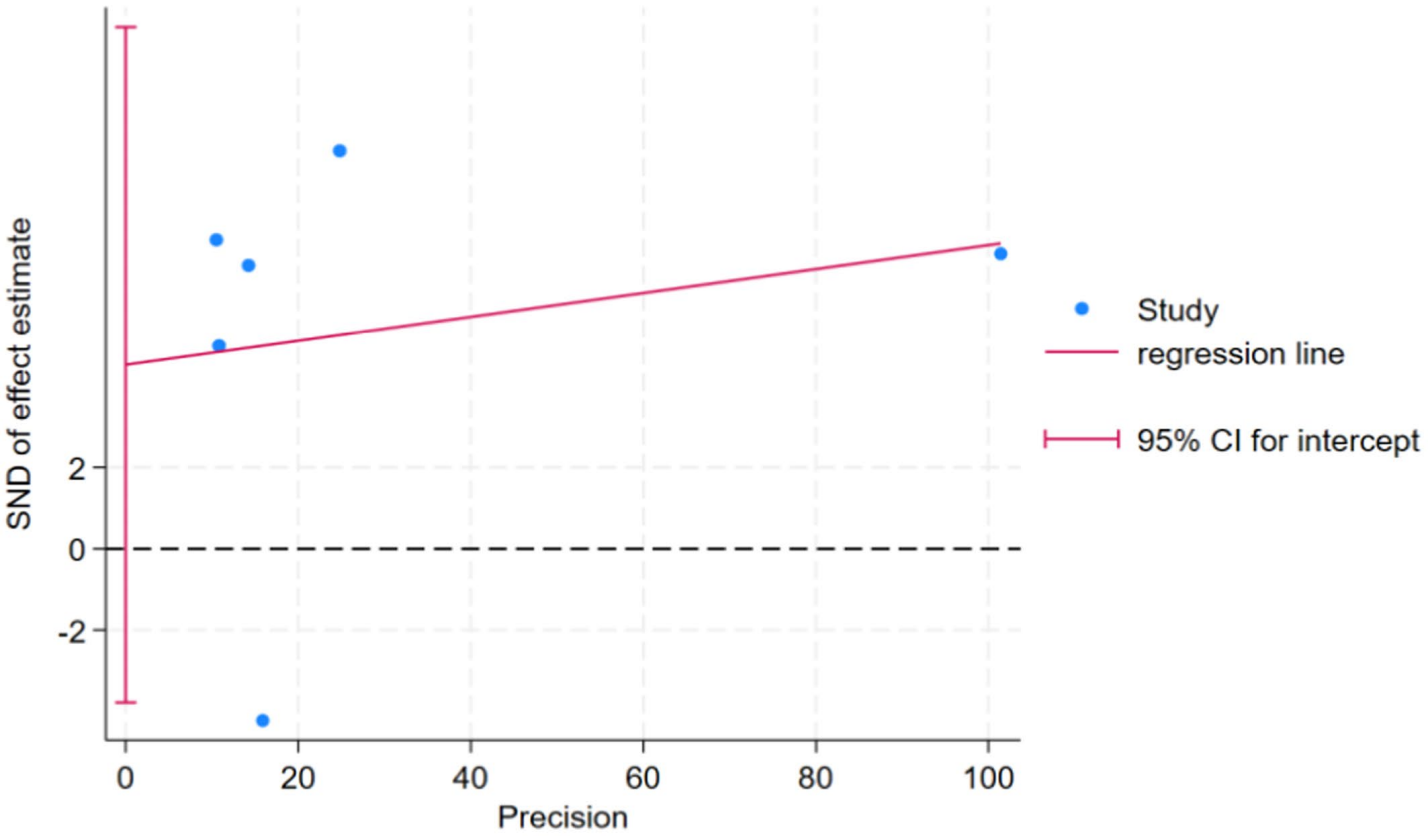
Publication bias (Egger’s Test).

Six case–control studies reported the risk of CKD in patients with IBD compared with a control group, finding that the risk of CKD was significantly higher in patients with than without IBD had (OR = 1.36, 95% CI: 1.08–1.70, *p* = 0.008, *I*^2^ = 97.3%, heterogeneity *p* < 0.00) (Figure 6). Because *I*^2^ was >50%, this analysis utilized a random-effects model (M-H heterogeneity) and performed sensitivity analysis (Figure 7) by systematically excluding individual studies to evaluate their impact on the combined effect. After excluding any individual study, the combined results of the remaining studies were not statistically significant, indicating that the original meta-analysis results could easily change significantly and lacked stability due to variations in the number of studies. Egger’s test found no publication bias (*p* = 0.205) (Figure 8).

**Figure 9:**
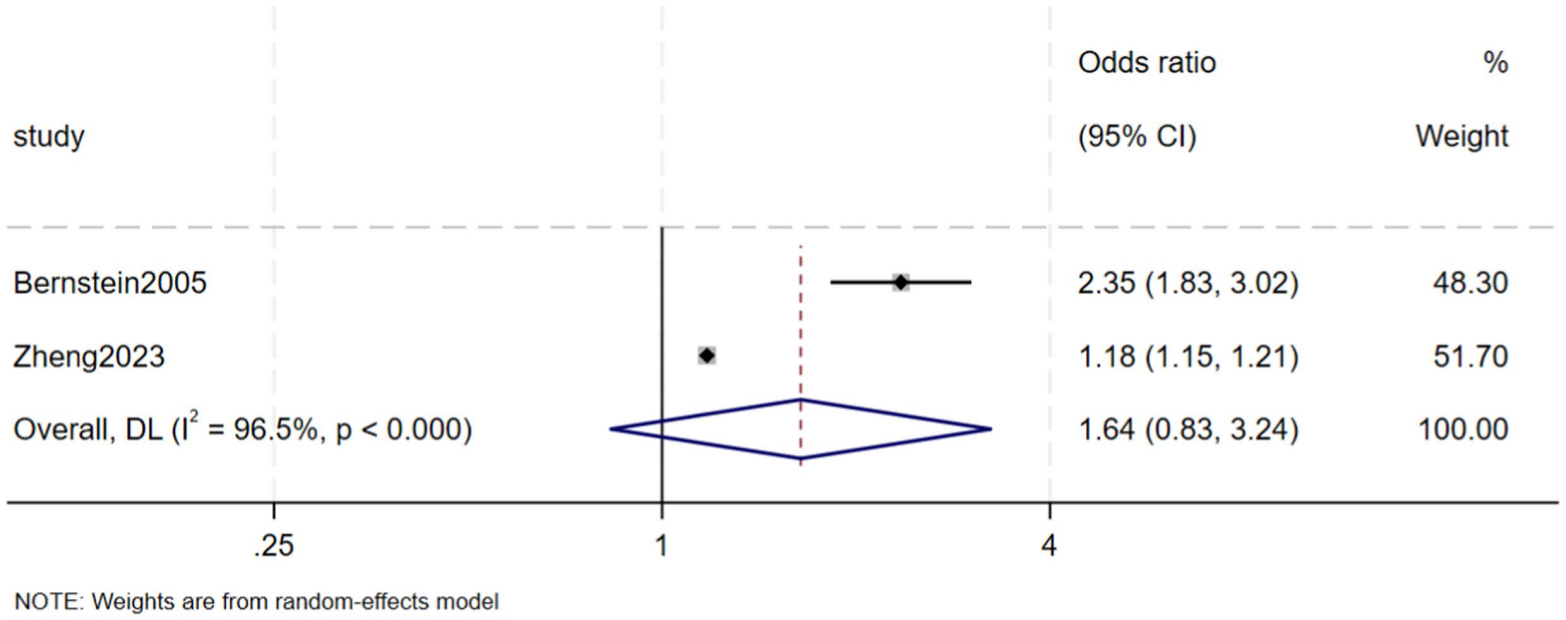
Risk of chronic kidney disease among the Crohn disease patients.

**Figure 10:**
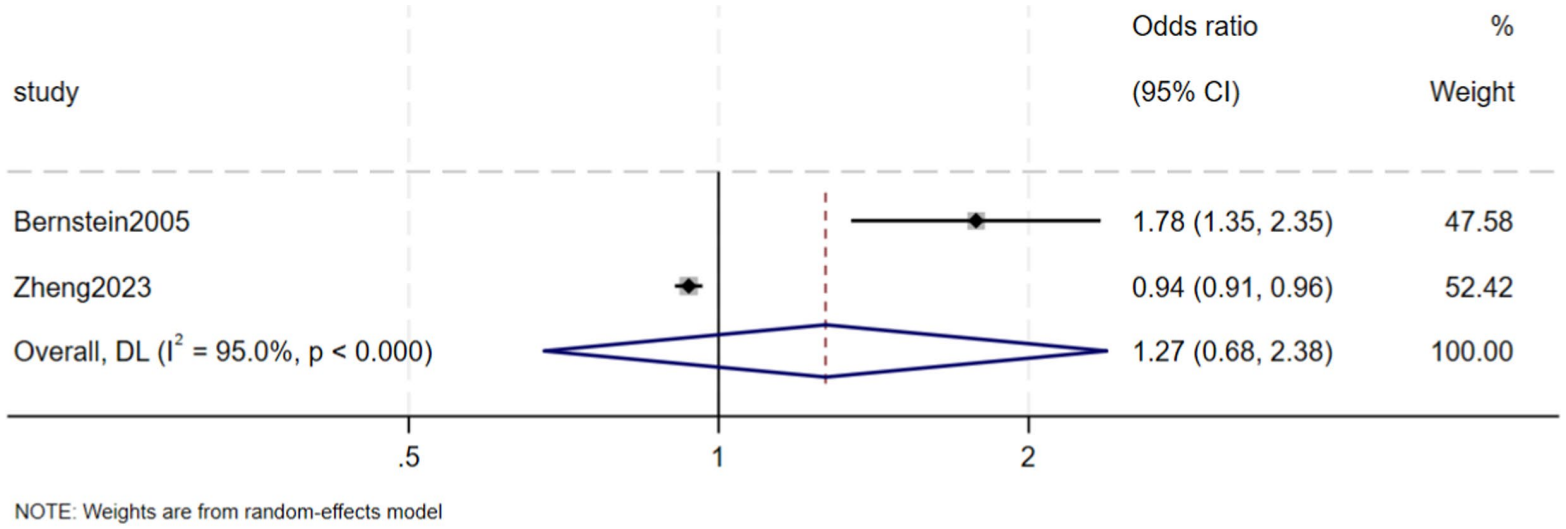
Risk of chronic kidney disease among the ulcerative colitis.

Two case–control studies separately reported the risk of CKD in CD and UC patients compared with a control group. Compared with control subjects, the ORs for CKD in CD and UC patients were 1.64 (95% CI 0.83–3.24, *p* = 0.151, *I*^2^ = 96.5%, heterogeneity *p* < 0.00) (Figure 9) and 1.27 (95% CI 0.68–2.38, *p* = 0.457, *I*^2^ = 95.0%, heterogeneity *p* < 0.00) (Figure 10), respectively.

Because only one case–control study reported the risk of ESRD in IBD patients compared with a control group, it was not subjected to a meta-analysis.

## 4 Discussion

This systematic review and meta-analysis found that the prevalence of CKD in patients with IBD was 5% (95% CI: 1–9%). Although the prevalence of CKD was higher in patients with CD (7%; 95% CI: −2–15%) than in patients with UC (6%; 95% CI: −2–15%), the difference was not statistically significant. The prevalence of ESRD in IBD patients was 0.2% (95% CI: −0.08–0.12%). The risk of CKD was found to be significantly higher in IBD patients than in a control group (OR = 1.36, 95% CI: 1.08–1.70, *p* = 0.008). Moreover, the ORs for CKD in CD and UC patients were 1.64 (95% CI: 0.83–3.24, *p* = 0.151) and 1.27 (95% CI: 0.68–2.38, *p* = 0.457), respectively.

Although studies have shown an increased risk of CKD in IBD, due to the small number of included studies and high heterogeneity across studies, it is not enough to definitively conclude that CKD is more common in patients with IBD. However, kidney function changes in IBD patients should still be of concern.

IBD is a systemic disease that primarily affects the intestines but is not limited to them. EIMs in IBD patients are caused by the same processes as intestinal inflammation but are located outside the intestines. The classic EIMs involve four organ systems: (1) the musculoskeletal system (e.g., arthritis and spondylitis); (2) the eyes (e.g., uveitis and episcleritis); (3) the skin (e.g., pyoderma gangrenosum, erythema nodosum, and oral ulcers); and (4) the liver-biliary system (e.g., primary sclerosing cholangitis) (12).

Although renal manifestations and complications are not rare in IBD patients, their direct correlation with intestinal diseases is not clear (13). Significant renal manifestations/complications in IBD include kidney stones, tubulointerstitial nephritis, glomerulonephritis, secondary amyloidosis, and medication-related complications (14), all of which can lead to impaired renal function, manifesting as acute or chronic renal insufficiency.

The risk of kidney stones has been reported higher in patients with than without IBD (15). Kidney stones are mainly caused by calcium oxalate or uric acid (16). The lifetime risk of kidney stones in IBD patients is approximately 18–20% (5), with this risk being higher in CD than in UC patients (5, 17). Patients with CD involving the colon are especially prone to oxalate stones, a condition known as enteric hyperoxaluria (EHO). EHO is thought to develop predominantly through three mechanisms: (1) increased absorption of oxalate salts, leading to increased excretion of oxalate salts in the urine and resulting in oxalate stone formation; (2) increased permeability of the colon to oxalate salts; and (3) decolonization of the gastrointestinal tract of IBD patients by the oxalate-degrading bacterium *Oxalobacter formigenes*, creating an ideal environment for promoting oxalate stone formation (18–20). Uric acid stones are common in patients with severe diarrhea or small intestine ostomies. The loss of intestinal fluid and bicarbonate leads to concentrated urine and increased acidity. Low urinary pH combined with low urine volume, especially in patients who have undergone colon surgery, promote the formation of uric acid stones (18, 21, 22). A single cohort study showed that recurrent urolithiasis was a major risk factor for renal insufficiency (23, 24). Therefore, complex IBD patients should be closely monitored, with regular urine analysis and upper urinary tract imaging (25).

Tubulointerstitial nephritis (TIN) in patients with IBD has been associated with exposure to 5-aminosalicylates (5-ASA) (26, 27), cyclosporine A, tumor necrosis factor-alpha (TNF-*α*) inhibitors (28) and vedolizumab (29). It is often challenging to determine whether renal dysfunction is a result of EIMs of IBD or drug therapy. Acute and chronic TIN have been reported to occur simultaneously with IBD, particularly CD, and in patients not exposed to nephrotoxic drugs (19). Kidney biopsies of untreated patients with CD have shown the presence of granulomas without eosinophilic infiltration. The co-occurrence in kidney biopsies of predominant lymphocytic infiltration with non-necrotizing granulomas further supports the diagnosis of TIN secondary to CD rather than drug-induced manifestations (30). Furthermore, TIN is most commonly associated with active intestinal disease, strongly suggesting that TIN is a true EIM of IBD (31, 32). Possible mechanisms of non-drug-related TIN include systemic immune dysregulation, cytokine activation, immune complexes targeting organ-specific epitopes shared by the colon and extracolonic sites (such as tubular basement membrane), and molecular mimicry (19).

Renal glomerular involvement in patients with IBD may be due to increased mucosal permeability, the deposition of intestinal-origin immune complexes following antigen exposure and the effects of drug therapy. The presence or worsening of glomerulonephritis has been reported consistent with the activity of intestinal diseases, indicating that renal glomerular injury is an EIM of IBD (19). Patients with IBD may have different histological types of glomerulonephritis, including IgA nephropathy, IgM nephropathy, membranous nephropathy, small vessel vasculitis, focal segmental glomerulosclerosis and anti-glomerular basement membrane glomerulonephritis (33). The most common diagnosis following kidney biopsy of these patients is IgA nephropathy, with the prevalence of IgA nephropathy being significantly higher than in kidney biopsy samples of all non-IBD patients (33, 34). Secondary IgA nephropathy in IBD may be a complex interaction involving mucosal inflammation, antigen rejection, chronic immune stimulation, and dysregulation of IgA production and transport (19). Furthermore, whole-genome association studies of IgA nephropathy have suggested a genetic cross-susceptibility between IBD and glomerular diseases (35). For example, HLA-DR1 has been associated with an increased risk of IgA nephropathy, whereas HLA-DR1/DQw5 has been associated with an increased risk of CD. Several loci associated with IgA nephropathy, such as CARD9 and HORMAD2, have also been associated with the risk of IBD. Other loci associated with IgA nephropathy, such as such as DEFA, TNFSF 13, VAV 3, ITGAM-ITGAX, and PSMB 8, encode proteins involved in maintaining the intestinal mucosal barrier or regulating mucosal immune responses (21). To date, however, there is no definitive information regarding the pathophysiology of other histological types of glomerulonephritis, such as IgM nephropathy, membranous nephropathy, small vessel vasculitis, focal segmental glomerulosclerosis, and anti-glomerular basement membrane glomerulonephritis (31).

Secondary amyloidosis (AA) is a rare but important complication of IBD. The most common clinical manifestation of AA is renal amyloidosis, typically presenting as proteinuria and nephrotic syndrome, which may progress to kidney failure (36). AA involves the deposition of amyloid fibrils derived from serum amyloid A (SAA) protein, an acute-phase reactant protein induced by chronic inflammation or infectious diseases that serves as a precursor to tissue amyloid proteins (37). In patients with CD and CD, the levels of SAA and acute-phase proteins correlate with disease activity (18). Early diagnosis of renal amyloidosis in IBD can improve patient prognosis.

Among the medications used to treat IBD, corticosteroids, azathioprine, metronidazole, low-dose methotrexate, and others have minimal or no renal toxicity (38). In contrast, ASAs, cyclosporine, tacrolimus, and TNF-*α* inhibitors may contribute to renal function impairment. However, the mechanisms by which these drugs participate in renal damage are generally unclear, making it difficult to determine whether renal impairment is due to EIMs or adverse drug reactions (31).

The ability of ASAs to cause renal damage is also unclear. Ulfasalazine, mesalazine, and olsalazine have been reported to induce renal toxicity, with the most common kidney alteration being interstitial nephritis (39). Systemic type I hypersensitivity reactions with fever and eosinophilia have also been observed (40).

Cyclosporine and tacrolimus are calcineurin inhibitors with immunosuppressive properties. The use of these drugs is often limited by severe adverse reactions, especially renal toxicity. Renal toxicity during treatment depends on the duration and dosage of treatment. The main features of chronic kidney damage are irreversible interstitial fibrosis and changes in small arteries (31).

TNF-*α* inhibitors, (including infliximab, adalimumab, certolizumab pegol, and golimumab) are increasingly used in the treatment of IBD (21). The anti-TNF-α produced by glomerular epithelial cells in patients with glomerulonephritis can lead to abnormal mucosal immune responses, such as the deposition of immune complexes of the drug itself or anti-drug antibodies, which can induce cell apoptosis and impair kidney function. Anti-TNF-α therapy can also shift the immune system to Th-2, upregulating the production of antibodies, including anti-nuclear, anti-dsDNA, and anti-neutrophil cytoplasmic antibodies, which may lead to the development of lupus-like and/or crescentic glomerulonephritis in susceptible individuals. Anti-TNF therapy can also increase the likelihood of viral or bacterial infections, which may promote different autoimmune reactions, such as molecular mimicry, bystander activation, or epitope spreading. In addition to anti-TNF drugs, other biologics that can cause this paradoxical effect. For example, adalimumab treatment of patients with refractory CD can lead to the development of tubulointerstitial nephritis (5, 31).

To our knowledge, this is the first study to analyze the incidence and risk of concurrent CKD in patients with IBD patients. However, this study had certain limitations. First, the number of studies included in the meta-analysis was limited, and there was significant heterogeneity among these studies, leading to the meta-analysis having insufficient robustness. Second, due to the limited number of studies included, this study did not perform stratified analyses based on factors such as patient gender and age.

## 5 Conclusion

Although studies have shown an increased risk of CKD in IBD, due to the small number of included studies and high heterogeneity across studies, it is not enough to definitively conclude that CKD is more common in patients with IBD. However, kidney function changes in IBD patients should still be of concern. Regular monitoring is crucial for early detection of kidney disease and prevention of progression to CKD. Early systematic diagnosis and management are recommended to reduce the burden of disease.

## Data Availability

The original contributions presented in the study are included in the article/Supplementary material, further inquiries can be directed to the corresponding author.
