# Supplementary material for: Concurrent chronic kidney disease in patients with inflammatory bowel disease, a systematic review and meta-analysis
Source: Front Med (Lausanne). 2024 Oct 3;11:1485087. doi: 10.3389/fmed.2024.1485087 (PMC11484269; doi:10.3389/fmed.2024.1485087)
Supplement: Supplementary file 1 [file Table_1.DOCX]

| Database | Search Terms | Results |
| --- | --- | --- |
| PubMed | (((((("Inflammatory Bowel Diseases"[Mesh]) OR (Inflammatory Bowel Diseases[Title/Abstract])) OR (Inflammatory Bowel Disease[Title/Abstract])) OR (Bowel Diseases, Inflammatory[Title/Abstract])) OR (((((("Colitis, Ulcerative"[Mesh]) OR (Colitis, Ulcerative[Title/Abstract])) OR (Idiopathic Proctocolitis[Title/Abstract])) OR (Ulcerative Colitis[Title/Abstract])) OR (Colitis Gravis[Title/Abstract])) OR (Inflammatory Bowel Disease, Ulcerative Colitis Type[Title/Abstract]))) OR (((((((((((((((((("Crohn Disease"[Mesh]) OR (Crohn Disease[Title/Abstract])) OR (Crohn's Enteritis[Title/Abstract])) OR (Regional Enteritis[Title/Abstract])) OR (Crohn's Disease[Title/Abstract])) OR (Crohns Disease[Title/Abstract])) OR (Inflammatory Bowel Disease 1[Title/Abstract])) OR (Enteritis, Granulomatous[Title/Abstract])) OR (Granulomatous Enteritis[Title/Abstract])) OR (Enteritis, Regional[Title/Abstract])) OR (Ileocolitis[Title/Abstract])) OR (Colitis, Granulomatous[Title/Abstract])) OR (Granulomatous Colitis[Title/Abstract])) OR (Ileitis, Terminal[Title/Abstract])) OR (Terminal Ileitis[Title/Abstract])) OR (Ileitis, Regional[Title/Abstract])) OR (Regional Ileitides[Title/Abstract])) OR (Regional Ileitis[Title/Abstract]))) AND ((((((((((((((((((("Kidney Failure, Chronic"[Mesh]) OR (Kidney Failure, Chronic[Title/Abstract])) OR (End-Stage Kidney Disease[Title/Abstract])) OR (Disease, End-Stage Kidney[Title/Abstract])) OR (End Stage Kidney Disease[Title/Abstract])) OR (Kidney Disease, End-Stage[Title/Abstract])) OR (Chronic Kidney Failure[Title/Abstract])) OR (End-Stage Renal Disease[Title/Abstract])) OR (Disease, End-Stage Renal[Title/Abstract])) OR (End Stage Renal Disease[Title/Abstract])) OR (Renal Disease, End-Stage[Title/Abstract])) OR (Renal Disease, End Stage[Title/Abstract])) OR (Renal Failure, End-Stage[Title/Abstract])) OR (End-Stage Renal Failure[Title/Abstract])) OR (Renal Failure, End Stage[Title/Abstract])) OR (Renal Failure, Chronic[Title/Abstract])) OR (Chronic Renal Failure[Title/Abstract])) OR (ESRD[Title/Abstract])) OR ((((((((((((((((((((("Renal Insufficiency, Chronic"[Mesh]) OR (Renal Insufficiency, Chronic[Title/Abstract])) OR (Chronic Renal Insufficiencies[Title/Abstract])) OR (Renal Insufficiencies, Chronic[Title/Abstract])) OR (Chronic Renal Insufficiency[Title/Abstract])) OR (Kidney Insufficiency, Chronic[Title/Abstract])) OR (Chronic Kidney Insufficiency[Title/Abstract])) OR (Chronic Kidney Insufficiencies[Title/Abstract])) OR (Kidney Insufficiencies, Chronic[Title/Abstract])) OR (Chronic Kidney Diseases[Title/Abstract])) OR (Chronic Kidney Disease[Title/Abstract])) OR (Disease, Chronic Kidney[Title/Abstract])) OR (Diseases, Chronic Kidney[Title/Abstract])) OR (Kidney Disease, Chronic[Title/Abstract])) OR (Kidney Diseases, Chronic[Title/Abstract])) OR (Chronic Renal Diseases[Title/Abstract])) OR (Chronic Renal Disease[Title/Abstract])) OR (Disease, Chronic Renal[Title/Abstract])) OR (Diseases, Chronic Renal[Title/Abstract])) OR (Renal Disease, Chronic[Title/Abstract])) OR (Renal Diseases, Chronic[Title/Abstract]))) | 465 |
| Web of Science | ((TS=(Renal Insufficiency, Chronic) OR AB=(Renal Insufficiency, Chronic OR Chronic Renal Insufficiencies OR Renal Insufficiencies, Chronic OR Chronic Renal Insufficiency OR Kidney Insufficiency, Chronic OR Chronic Kidney Insufficiency OR Chronic Kidney Insufficiencies OR Kidney Insufficiencies, Chronic OR Chronic Kidney Diseases OR Chronic Kidney Disease OR Disease, Chronic Kidney OR Diseases, Chronic Kidney OR Kidney Disease, Chronic OR Kidney Diseases, Chronic OR Chronic Renal Diseases OR Chronic Renal Disease OR Disease, Chronic Renal OR Diseases, Chronic Renal OR Renal Disease, Chronic OR Renal Diseases, Chronic)) OR (TS=(Kidney Failure, Chronic) OR AB=(Kidney Failure, Chronic OR End-Stage Kidney Disease OR Disease, End-Stage Kidney OR End Stage Kidney Disease OR Kidney Disease, End-Stage OR Chronic Kidney Failure OR End-Stage Renal Disease OR Disease, End-Stage Renal OR End Stage Renal Disease OR Renal Disease, End-Stage OR Renal Disease, End Stage OR Renal Failure, End-Stage OR End-Stage Renal Failure OR Renal Failure, End Stage OR Renal Failure, Chronic OR Chronic Renal Failure OR ESRD))) AND ((TS=(Inflammatory Bowel Diseases) OR AB=(Inflammatory Bowel Diseases OR Inflammatory Bowel Disease OR Bowel Diseases, Inflammatory)) OR (TS=(Colitis, Ulcerative) OR AB=(Colitis, Ulcerative OR Idiopathic Proctocolitis OR Ulcerative Colitis OR Colitis Gravis OR Inflammatory Bowel Disease, Ulcerative Colitis Type)) OR (TS=(Crohn Disease) OR AB=(Crohn Disease OR Crohn's Enteritis OR Regional Enteritis OR Crohn's Disease OR Crohns Disease OR Inflammatory Bowel Disease 1 OR Enteritis, Granulomatous OR Granulomatous Enteritis OR Enteritis, Regional OR Ileocolitis OR Colitis, Granulomatous OR Granulomatous Colitis OR Ileitis, Terminal OR Terminal Ileitis OR Ileitis, Regional OR Regional Ileitides OR Regional Ileitis))) | 791 |
| Embase | #8. #4 AND #7 634  #7. #5 OR #6 231,756  #6. 'kidney failure, chronic':ab,ti OR 'end-stage 118,644 kidney disease':ab,ti OR 'disease, end-stage  kidney':ab,ti OR 'end stage kidney disease':ab,ti  OR 'kidney disease, end-stage':ab,ti OR 'chronic  kidney failure':ab,ti OR 'end-stage renal  disease':ab,ti OR 'disease, end-stage  renal':ab,ti OR 'end stage renal disease':ab,ti  OR 'renal disease, end-stage':ab,ti OR 'renal  disease, end stage':ab,ti OR 'renal failure,  end-stage':ab,ti OR 'end-stage renal  failure':ab,ti OR 'renal failure, end  stage':ab,ti OR 'renal failure, chronic':ab,ti OR  'chronic renal failure':ab,ti OR esrd:ab,ti  #5. 'enal insufficiency, chronic':ab,ti OR 'chronic 130,094 renal insufficiencies':ab,ti OR 'renal  insufficiencies, chronic':ab,ti OR 'chronic renal  insufficiency':ab,ti OR 'kidney insufficiency,  chronic':ab,ti OR 'chronic kidney  insufficiency':ab,ti OR 'chronic kidney  insufficiencies':ab,ti OR 'kidney  insufficiencies, chronic':ab,ti OR 'chronic  kidney diseases':ab,ti OR 'chronic kidney  disease':ab,ti OR 'disease, chronic kidney':ab,ti  OR 'diseases, chronic kidney':ab,ti OR 'kidney  disease, chronic':ab,ti OR 'kidney diseases,  chronic':ab,ti OR 'chronic renal diseases':ab,ti  OR 'chronic renal disease':ab,ti OR 'disease,  chronic renal':ab,ti OR 'diseases, chronic  renal':ab,ti OR 'renal disease, chronic':ab,ti OR  'renal diseases, chronic':ab,ti  #4. #1 OR #2 OR #3 155,947  #3. 'crohn disease':ab,ti OR 'crohns enteritis':ab,ti 2023  OR 'regional enteritis':ab,ti OR 'crohns  disease':ab,ti OR 'inflammatory bowel disease  1':ab,ti OR 'enteritis, granulomatous':ab,ti OR  'granulomatous enteritis':ab,ti OR 'enteritis,  regional':ab,ti OR ileocolitis:ab,ti OR 'colitis,  granulomatous':ab,ti OR 'granulomatous  colitis':ab,ti OR 'ileitis, terminal':ab,ti OR  'terminal ileitis':ab,ti OR 'ileitis,  regional':ab,ti OR 'regional ileitides':ab,ti OR  'regional ileitis':ab,ti  #2. 'colitis, ulcerative':ab,ti OR 'idiopathic 80,661 proctocolitis':ab,ti OR 'ulcerative  colitis':ab,ti OR 'colitis gravis':ab,ti OR  'inflammatory bowel disease, ulcerative colitis  type':ab,ti  #1. 'inflammatory bowel diseases':ab,ti OR 104,608  'inflammatory bowel disease':ab,ti OR 'bowel  diseases, inflammatory':ab,ti | 634 |
| Cochrane Library | #1 MeSH descriptor: [Kidney Failure, Chronic] explode all trees 5550  #2 (Kidney Failure, Chronic OR End-Stage Kidney Disease OR Disease, End-Stage Kidney OR End Stage Kidney Disease OR Kidney Disease, End-Stage OR Chronic Kidney Failure OR End-Stage Renal Disease OR Disease, End-Stage Renal OR End Stage Renal Disease OR Renal Disease, End-Stage OR Renal Disease, End Stage OR Renal Failure, End-Stage OR End-Stage Renal Failure OR Renal Failure, End Stage OR Renal Failure, Chronic OR Chronic Renal Failure OR ESRD):ti,ab,kw (Word variations have been searched) 20061  #3 #1 or #2 20061  #4 MeSH descriptor: [Renal Insufficiency, Chronic] explode all trees 8653  #5 (Renal Insufficiency, Chronic OR Chronic Renal Insufficiencies OR Renal Insufficiencies, Chronic OR Chronic Renal Insufficiency OR Kidney Insufficiency, Chronic OR Chronic Kidney Insufficiency OR Chronic Kidney Insufficiencies OR Kidney Insufficiencies, Chronic OR Chronic Kidney Diseases OR Chronic Kidney Disease OR Disease, Chronic Kidney OR Diseases, Chronic Kidney OR Kidney Disease, Chronic OR Kidney Diseases, Chronic OR Chronic Renal Diseases OR Chronic Renal Disease OR Disease, Chronic Renal OR Diseases, Chronic Renal OR Renal Disease, Chronic OR Renal Diseases, Chronic):ti,ab,kw (Word variations have been searched) 19734  #6 #4 or #5 21792  #7 #3 or #6 27558  #8 MeSH descriptor: [Inflammatory Bowel Diseases] explode all trees 4872  #9 (Inflammatory Bowel Diseases OR Inflammatory Bowel Disease OR Bowel Diseases, Inflammatory):ti,ab,kw (Word variations have been searched) 4656  #10 #8 or #9 7886  #11 MeSH descriptor: [Colitis, Ulcerative] explode all trees 2037  #12 (Colitis, Ulcerative OR Idiopathic Proctocolitis OR Ulcerative Colitis OR Colitis Gravis OR Inflammatory Bowel Disease, Ulcerative Colitis Type):ti,ab,kw (Word variations have been searched) 6343  #13 #11 or #12 6343  #14 MeSH descriptor: [Crohn Disease] explode all trees 2547  #15 (Crohn Disease OR Crohn's Enteritis OR Regional Enteritis OR Crohn's Disease OR Crohns Disease OR Inflammatory Bowel Disease 1 OR Enteritis, Granulomatous OR Granulomatous Enteritis OR Enteritis, Regional OR Ileocolitis OR Colitis, Granulomatous OR Granulomatous Colitis OR Ileitis, Terminal OR Terminal Ileitis OR Ileitis, Regional OR Regional Ileitides OR Regional Ileitis):ti,ab,kw (Word variations have been searched) 9203  #16 #14 or #15 9203  #17 #10 or #13 or #16 14171  #18 #7 and #17 185 | 185 |
